# CHIP Decline Is Associated With Isoflurane-Induced Neurodegeneration in Aged Mice

**DOI:** 10.3389/fnins.2022.824871

**Published:** 2022-03-18

**Authors:** Qiaoqiao Xu, Juan Xiong, Li Xu, Yuanyuan Wu, Man Li, Qinqin Li, Tao Jiang, Ailin Luo, Yi Zhang

**Affiliations:** ^1^Department of Anesthesiology, Tongji Hospital, Tongji Medical College, Huazhong University of Science and Technology, Wuhan, China; ^2^Department of Anesthesiology, Wuhan Children’s Hospital (Wuhan Maternal and Child Healthcare Hospital), Tongji Medical College, Huazhong University of Science and Technology, Wuhan, China

**Keywords:** PND, isoflurane, neurodegeneration, CHIP, synaptic function

## Abstract

Perioperative neurocognitive disorders (PND) commonly occur in elderly patients, and isoflurane could be a risk factor. During the pathogenesis of neurodegeneration, the ubiquitin–proteasome system (UPS) participates in the process of aging, which affects synaptic plasticity and synaptic function. However, whether UPS is involved in the etiology of PND is unclear. In this study, we examined the expression change of ubiquitin E3 ligase protein carboxyl-terminus of Hsc70-interacting protein (CHIP) and the function turbulence of UPS in isoflurane-exposed aged mouse to illustrate the role of UPS in PND. Neurodegenerative behavioral changes were shown in isoflurane-exposed aged mice and correlated with neuropathological changes manifested with reduced number of intersections and spine density in the cortex. Ubiquitin function was decreased while the apoptosis was activated, and CHIP protein expression decline altered synapsin expression and phosphorylation associated with the neurodegeneration in isoflurane-induced PND. Aging was the big important factor. And it remained consistent with the synapsin phosphorylation/dephosphorylation level changes in CHIP knock-down N2a cells. Per our observation, the decline in CHIP protein expression and synaptic degeneration might reveal the reason for synaptic degeneration in the underlying pathogenesis of PND caused by isoflurane.

## Introduction

Perioperative neurocognitive disorders (PND) were characterized as a decline in cognitive functions, including attention, memory, orientation, executive function, and language fluency, which was associated with increased morbidity and mortality (Lin et al., 2012; Skvarc et al., 2018). Aging increased the susceptibility of PND (Lin et al., 2012). About 10% of elderly (60 years or older) patients developed PND 3 months after non-cardiac surgery (Monk et al., 2008). Most surgeries had been maintained with inhalation anesthetics, i.e., isoflurane and sevoflurane (Monk et al., 2008). Isoflurane, a commonly used inhalation anesthetic, had been suspected to increase the incidence of PND (Simon et al., 2001).

Neuronal damage was concomitant with an elevation of neurodegeneration markers, which was of particular importance in elderly subjects (Lewczuk et al., 2018; DiMeglio et al., 2019). The proteins and molecular chaperones in the ubiquitin–proteasome system (UPS) were crucial for intracellular protein homeostasis and degradation of aberrant and damaged proteins and affected the synaptic plasticity and function. It had been implicated in the pathogenesis of many neurodegenerative diseases (Wang et al., 2020).

It had been reported that sevoflurane acts on the ubiquitination–proteasome pathway to facilitate post-synaptic density 95 (PSD95) protein degradation (Lu et al., 2017). It indicated that inhalation anesthetics may target the UPS to regulate synaptic function, which could be a plausible reason for PND. Nonetheless, whether isoflurane anesthesia disturbed the UPS in aging and what its role in PND is have not been elucidated yet.

A U-box type chaperone, the carboxyl-terminus of Hsc70-interacting protein (CHIP), was associated with ubiquitin E3 ligase, which was responsible for catalyzing the transfer of ubiquitin to substrate proteins (Paul and Ghosh, 2014). CHIP had been reported to exert multiple functions, such as protein degradation, signal transduction, apoptosis, and autophagy (Ferreira et al., 2013; Guo et al., 2015; Tawo et al., 2017; Hwang et al., 2018). Therefore, we hypothesized that CHIP protein with ubiquitin E3 ligase activity may be involved in the mechanism of PND in aging. In this study, we investigated the behavioral changes and brain tissue neuropathological changes in PND induced by isoflurane in aged mice. We further examined the association between CHIP protein expression and function changes and then attempted to reveal the reason for synaptic degeneration in the underlying pathogenesis of PND caused by inhalation anesthetic.

## Materials and Methods

### Animals and Housing

The study protocols were approved by the experimental animal Committee at Tongji Hospital, Tongji Medical College, Huazhong University of Science and Technology. The maintenance and handling of mice were performed in accordance with the National Institute of Health Guide for the Care and Use of Laboratory Animals. The mice were housed and bred in the animal facilities under a 12-h light and 12-h dark cycle and temperature-controlled (22 ± 2°C) conditions. *Ad libitum* access to food and water was provided.

### Isoflurane Exposure and Experimental Timeline

A total of 80 C57 male mice at the age of 12 months were purchased from the Laboratory Animal Center of Tongji Hospital, Tongji Medical College, Huazhong University of Science and Technology. These mice needed 2 weeks to acclimatize. After that, the preliminary experiment was accomplished. The 12.5-month-old mice were considered as middle-aged mice. And when the age was up to 15 months, these mice were considered to be in their early elderly stage (Lin et al., 2012). Therefore, mice used in this study were randomly divided into CON group (no isoflurane exposure), ISO-12.5 group (isoflurane exposure at 12.5 months of age), and ISO-15 group (isoflurane exposure at 15 months of age). At the age of 12.5 or 15 months, mice were placed in a temperature-controlled chamber with a heated pad to maintain the experimental temperature at 37°C before isoflurane or control gas exposure as we described previously (Tang et al., 2020). Mice in the control group received 60% oxygen (balanced with air) in a chamber for 6 h, while mice subjected to isoflurane exposures were placed in a similar anesthesia chamber for 6 h and exposed to 2% isoflurane flushed with 60% oxygen (balanced with air) for 2 h. The homeothermic chambers sizes were 20 cm × 20 cm × 10 cm. After the treatment, the mice were placed back in their home cages (*n* = 10 per group). All the mice were subjected to behavior tests 1 week later and to be perfused for Golgi staining and western blotting 1 month later.

### Behavior Tests

#### Morris Water Maze

The Morris water maze (MWM) (*n* = 10 per group) was placed in a single room, with four graphic signals hung on the walls. A round and steel pool (diameter, 150 cm; height, 60 cm) was filled up with water until the water level reached 1.0 cm over the level of a platform (diametric distance, 10 cm). The training protocol for the task of the MWM test had three trials (60 s maximum; interval 30 min) each day and lasted for five consecutive days. After 2 h at the end of the fifth day of training, the probe trial was performed. After a 1-day interval, the probe trial was performed again without training on the seventh day, and in this way the long-term cognition memory of mice could be tested. AVTAS v3.3 (Ani Lab Software and Instruments Co., Ltd., Ningbo, China), an automated Journal Pre-proof 10 video-tracking system, was used to track the motion of mice in the pool. The time spent searching and mounting the platform (latency), the path length, and the duration of time spent in each quadrant and platform crossing were calculated and determined.

#### Footprint

Mice were allowed to walk across a paper-lined chamber and into an enclosed box (*n* = 10 per group). After one practice run, tracings for front and rear footprints for each mouse were measured. Measurements were averaged, and the data were presented as stride length in centimeters.

#### Light–Dark Box

The light–dark test (*n* = 10 per group) used the apparatus (length 45 cm, width 24 cm, and height 21 cm) consisting of two equal acrylic compartments, one dark and one light, which were separated by a divider with a 5 cm × 7 cm opening at floor level. The light area was illuminated by a 60-W bulb lamp. A mouse was placed in the center of the light area and allowed to explore the novel environment for 5 min. During the next 5 min, the number of transfers between the two compartments and the time staying in the light area were recorded.

### Golgi Staining

Mice were anesthetized with chloral hydrate (i.p., 300 mg kg^–1^) and sacrificed by decapitation (*n* = 6 per group). The forebrain was placed in lightproof glass jars with 40 ml of Golgi–Cox solution (5% potassium dichromate; 5% mercuric chloride; 5% potassium chromate, in distilled water) for 2 days at 37°C and then transferred into protecting solution at 4°C in the dark (Zaqout and Kaindl, 2016). Coronal sections (150 μm) were collected. The Golgi sections were visualized with ammonium hydroxide and dehydrated through a series of graded ethanol and xylene, and then mounted on slides and cover slipped for viewing. To minimize bias, the experimenter remained blind to the treatment conditions throughout the procedure. Cortical pyramidal neurons were traced with a light microscope (Leica, DM6, 209 objective lens). Sholl analysis was employed by Image J and Neuron Studio software to assess the morphological structure of dendrites (Janthakhin et al., 2017). These second-order basilar dendritic branch (>30 μm, five segments of each mouse) were arbitrarily selected in basal and apical dendrites. Dendritic spines were counted using Image J, and density was expressed as the number of spines per 20 μm.

### Western Blotting

Cultured cells and brain tissues (*n* = 6 per group) from the cortex and hippocampus were homogenized at 4°C in radioimmunoprecipitation assay buffer [50 mM Tris, pH 8.0, 150 mM NaCl, 1 mM EDTA, pH 8.0, 1 mM EGTA, pH 8.0, 0.1% sodium dodecyl sulfate (SDS), 0.5% deoxycholate, and 1% Triton X-100] with protease inhibitor (P8340; Sigma Aldrich). The homogenate was centrifuged at 10,000 *g* for 15 min at 4°C, and the supernatant was diluted in SDS sample buffer (62.6 mM Tris–HCl, pH 6.8, 2% SDS, 10% glycerol, and 0.01% bromophenol blue) and sonicated for 10 s after incubation at 100°C for 5 min. The lysates were subjected to 10% SDS–polyacrylamide gel electrophoresis. Proteins were transferred to nitrocellulose membrane. After blocking non-specific sites with Tris-buffered saline (TBS) containing 5% defatted dried milk, membranes were probed with the antibodies. Anti-β-actin mouse antibody (Affinity) blotting was used as a loading control. Rabbit antibodies were CHIP (proteintech), synapsin I (SYN I, proteintech), phosphorylated synapsin I S9 (SYN I-S9, Affinity), S427 (SYN I-S427, Affinity), S605 (SYN I-S605, Affinity), ubiquitin (proteintech), SNAP25 (Affinity), and PSD95 (Affinity).

### Analysis of Ubiquitin–Proteasome System and Caspase-3 Activity

For UPS and caspase-3 activity detection, brain tissues from the cortex and hippocampus were lysed (*n* = 6 per group). According to the manufacturer protocols, the tissues were subjected to UPS activity measurement by using E3/UBPL assay kit (MSKBIO KT86452) and caspase-3 activity measurement by using caspase-3 assay kit (MSKBIO KT21353).

### Cell Culture and SiRNA Transfection

N2a cells were cultured in Dulbecco’s modified essential medium (DMEM) medium supplied with 10% fetal bovine serum (FBS), penicillin (100 U ml^–1^), and streptomycin (100 mg ml^–1^). Cells were plated 24 h before transfection with siRNA (5 μl in six-well plate according to the manufacturer protocols from Ribobio) by Lipofectamine 2000 (Thermo Fisher Scientific). Stub1 was the gene name of CHIP. Three Stub1-siRNA were used: (① CTGGAACAGTATCGAGGAA, ② CAACTTTGGGG ATGATATT, and ③ GGAGATGGAGAGTTATGAT). The medium was changed 6 h later, and cells were harvested after 48 h.

### Statistical Analysis

Unless stated otherwise, values in the figures and text were presented as means ± SEM. Statistical analysis used Student’s *t*-test (two tailed) for comparing two different groups. For behavioral data analysis, we used one-way analysis of variance, which was followed by Tukey’s honestly significant difference *post-hoc* test. A value of *p* < 0.05 was considered to be statistically significant, and levels of significance were defined as *p < 0.05, ^**^p < 0.01, and ^***^p < 0.001.

## Results

### The Neurodegenerative Behavioral Changes Were Shown in Isoflurane-Exposed Aged Mice

**FIGURE 1:**
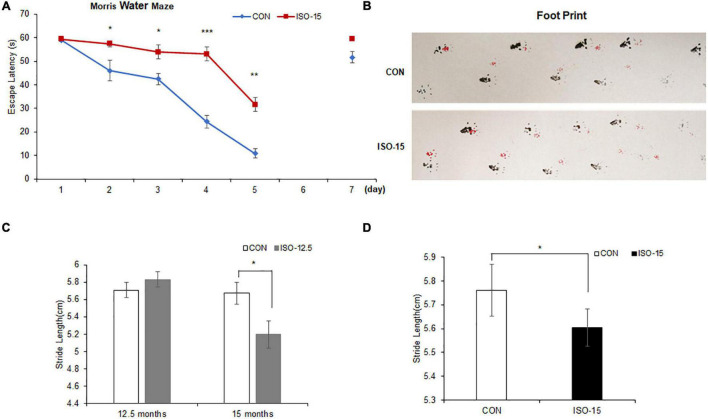
The spatial learning cognitive impairment and motor dysfunction were reduced in isoflurane-exposed aged mice: **(A)** 15-month-old mice exposed to isoflurane displayed prolonged escape latency compared with the CON group from the second test day to the fifth test day, while 1 day no test interval later, the difference between the two groups was not significant (*n* = 10, *p < 0.05, **p < 0.01, ***p < 0.001). **(B)** The footprint of mice in different groups. **(C)** At the age of 12.5 months, there were no significant differences in the gait function between the two groups. However, until these mice were at the age of 15 months, the stride length of those mice exposed to 2% isoflurane was significantly lower than that in the CON group (*n* = 10, *p < 0.05). **(D)** The stride length of 15-month-old mice exposed to 2% isoflurane was significantly lower than that in the CON group (*n* = 10, *p < 0.05).

To verify the influence of isoflurane (2%, 2 h) exposure on spatial and long-term cognition, the MWM task was performed. Fifteen-month-old mice exposed to isoflurane displayed spatial learning cognitive impairment in MWM as compared with the CON group, which showed prolonged escape latency (Figure 1A). After 1-day interval to test the long-term cognition memory, it did not differ significantly between the two groups on the seventh day (Figure 1A).

We tested the motor performance of mice under exposure of 2% isoflurane at different age by footprint. At the age of 12.5 months, there was no significant difference on the gait function between the ISO-12.5 group and the CON group. However, when the ISO-12.5 group reached the age of 15 months, the gait function was assessed again, and it was shown that the average stride length was significantly lower than that in the CON group (Figure 1B). Moreover, a similar impaired motor function was also found in the mice in the ISO-15 group (Figures 1C,D).

**FIGURE 2:**
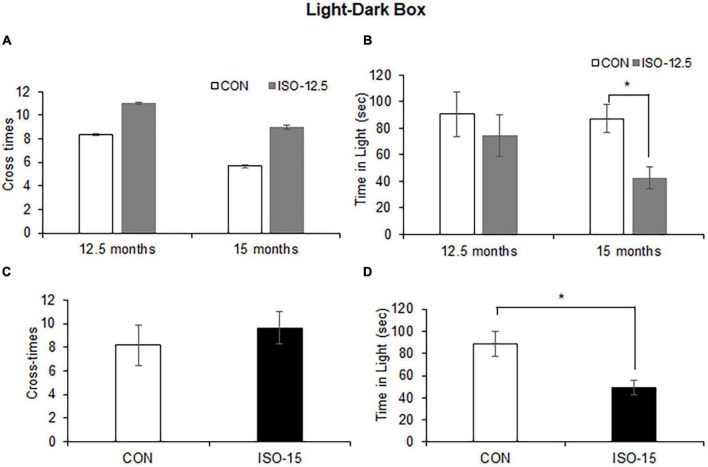
Anxiety was shown in isoflurane-exposed old mice (*n* = 10). **(A)** The cross-time differences between the ISO-12.5 group and the CON group were not significant at age of 12.5 or 15 months. **(B)** The time stayed in the light box revealed not significant difference between the ISO-12.5 group and the CON group; however, a significant reduction of time stayed in the light box was shown in the ISO-12.5 group when these mice were at the age of 15 months (*p < 0.05). **(C)** The cross-time difference between the ISO-15 group and the CON group were not significant. **(D)** The time stayed in the light box was significantly reduced in the ISO-15 group compared with the CON group (*p < 0.05).

The light–dark box test assessed anxiety-like behaviors by quantifying general locomotor activity and willingness to explore. The cross-time differences between the ISO-12.5 group and the CON group were not significant at the age of 12.5 months or up to 15 months (Figure 2A). And the difference in the time stayed in the light box was not significant between the ISO-12.5 group and the CON group at the age of 12.5 months. However, a significant reduction was shown in the mice in the ISO-12.5 group when they reached the age of 15 months (Figure 2B). Additionally, in the ISO-15 group mice a significant reduction in the time stayed in the light box was manifested with no cross-time differences (Figures 2C,D), suggesting increased anxiety-like behavior.

### The Intersection Number and Spine Density of the Neuron in the Cortex Were Reduced in Isoflurane-Exposed Aged Mice

**FIGURE 3:**
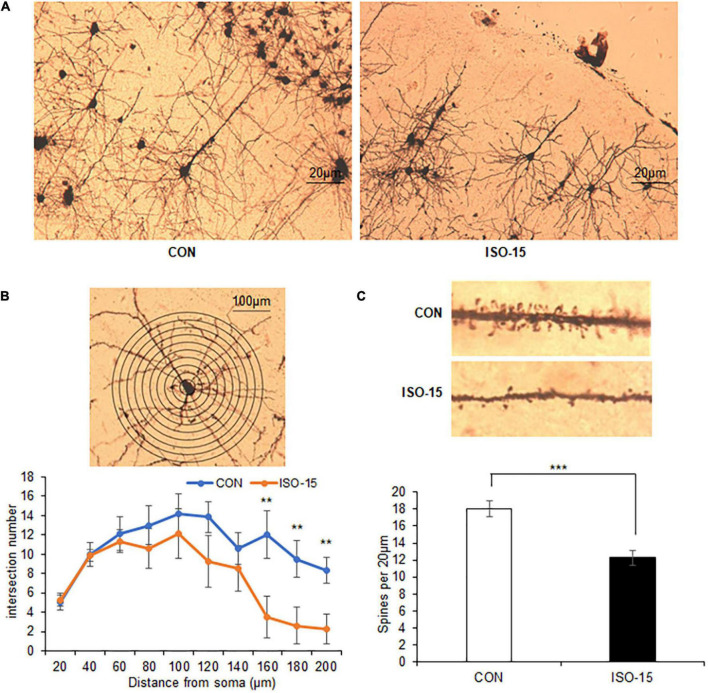
The intersection number and spine density of the neuron in the cortex were reduced in isoflurane-exposed aged mice. **(A)** The Golgi staining picture to show neuron in the cortex in different groups. **(B)** The number of intersections at 150–200 μm from the cell soma was reduced in the cortex in the ISO-15 group compared with the CON group (*n* = 6, **p < 0.01). **(C)** The spine density was reduced in the cortex in the ISO-15 group compared with the CON group (*n* = 6, ***p < 0.001).

It had been reported that isoflurane does not alter dendritic spine in the adult mouse cortex (Antila et al., 2017). However, the isoflurane impacts on aged mice remained unclear. In order to assess whether isoflurane treatment might alter the synaptic structure, we did immunohistology analysis. Reduced intersections at 150–200 μm from the cell soma of the cortex neuron were found in the ISO-15 group mice (Figures 3A,B). Extremely significant decrease in spine density in the cortex neuron was revealed as well (Figure 3C). These results demonstrated that isoflurane exposure could cause morphological changes in the neurons of aged mice cortex, which could be the reason for the changes of behavior and performance.

### Impaired Ubiquitin–Protease Pathway With Concomitant Activation of Apoptosis in the Isoflurane-Exposed Aged Mice

**FIGURE 4:**
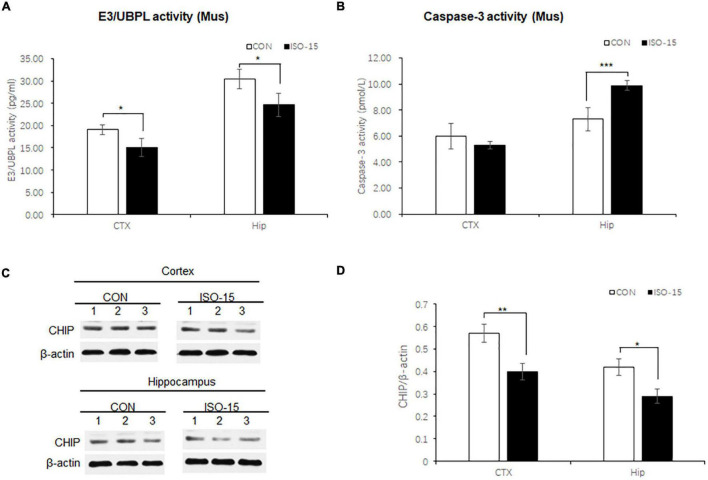
Ubiquitin function was decreased with Ubiquitin E3 ligase CHIP expression decline, and the apoptosis was activated in isoflurane-exposed aged mice. **(A)** The E3/UBPL activities in the cortex and hippocampus brain region were decreased in the ISO-15 group (*n* = 6, *p < 0.05). **(B)** The caspase-3 activity was increased in the hippocampus in the ISO-15 group (*n* = 6, *p < 0.05, ***p < 0.001). **(C)** CHIP expression was decreased in the cortex and hippocampus brain tissue in the ISO-15 group. **(D)** Statistical bar chart to show that CHIP expression was present (*n* = 6, *p < 0.05, **p < 0.01).

The UPS participated in the process of aging-related neurodegeneration, affected the synaptic plasticity and synaptic function, and regulated caspase-3 activity in the apoptosis pathway (Zhang et al., 2010; Kumar et al., 2018). To detect the UPS and caspase-3 activity, brain tissues lysates from the cortex and hippocampus were collected to measure the E3/UBPL and caspase-3 activity. E3/UBPL activities were decreased in the cortex and hippocampus (Figure 4A), and caspase-3 activity was increased in the hippocampus in ISO-15 mice (Figure 4B). It indicated that after the 15-month-old mice received isoflurane exposure, the ubiquitin function was reduced accompanied with the apoptosis pathway activation.

### Ubiquitin E3 Ligase and Synaptic Protein Expression Changed in Isoflurane-Exposed Aged Mice

**FIGURE 5:**
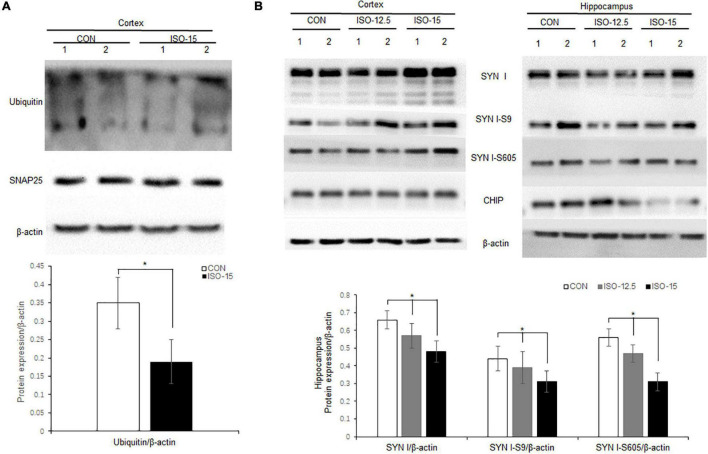
Ubiquitin and synaptic protein expression changed in isoflurane-exposed aged mice. **(A)** Ubiquitin expression was decreased in the cortex brain tissue in the ISO-15 group, while there was no significant difference in synaptic protein SNAP25 expression in the cortex (*n* = 6, *p < 0.05). **(B)** In the brain tissue of cortex, there was no significant difference in the expression level of synapsin I and the phosphorylation level of synapsin I S9 and S605 between CON group, ISO-12.5 group, and ISO-15 group. In the brain tissue of the hippocampus, the expression level of synapsin I and the phosphorylation level of synapsin I S9 and S605 were decreased in both the ISO-12.5 group and ISO-15 group, compared with the CON group, and the expression level of CHIP had a dramatic decline in the ISO-15 group (*n* = 6, *p < 0.05).

**Neurodegenerative** behavioral and neuropathological changes implied synaptic degeneration in isoflurane-exposed aged mice, which might be associated with ubiquitin function decrease. We evaluated the ubiquitin E3 ligase CHIP and synaptic protein expression in the mice brain. Our data revealed that the expression of CHIP was decreased in the cortex and hippocampus tissue in those mice in the ISO-15 group (Figures 4C,D). Since the ubiquitin E3 expression declined, there was less protein in the ubiquitination; then a reduced level of ubiquitin was found in the cortex, while there was no significant difference in the expression of another important synaptic protein, SNAP25, in the cortex (Figure 5A). Furthermore, we compared the synaptic protein expression level in the 15-month-old mice among different isoflurane exposure time groups. In the brain tissue of the cortex, there was no significant difference in the expression level of synapsin I and the phosphorylation level of synapsin I-S9 or S605 between the CON group and ISO-12.5 group, as well as the CHIP expression level (Figure 5B). And there was no statistical significance in the expression level of synapsin I and the phosphorylation level of synapsin I S9 and S605 in ISO-15 group in the cortex, while the CHIP expression level was decreased (Figure 5B). However, in the brain tissue of the hippocampus, the expression level of synapsin I and the phosphorylation level of synapsin I-S9 and S605 were decreased in both the ISO-12.5 group and ISO-15 group, compared with CON group, and the expression level of CHIP had a dramatic decline in the ISO-15 group (Figure 5B).

### Decreased CHIP Expression Altered Synaptic Protein Expression and Phosphorylation in vitro

**FIGURE 6:**
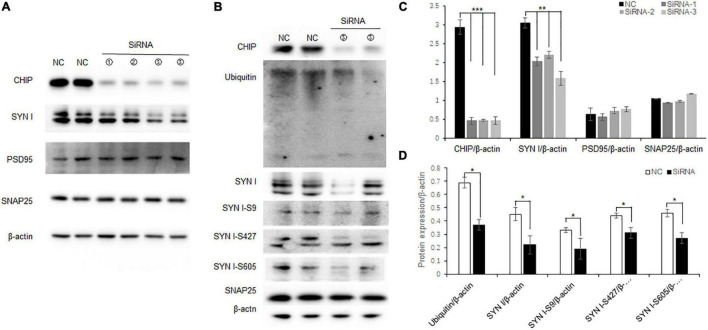
Decreased CHIP expression altered synaptic protein expression and phosphorylation in N2a cells. **(A)** CHIP knock-down protein was designed by siRNA in N2a cells. Approximately 5 μl Stub1-siRNA was able to achieve effective knock-down CHIP expression, and consequently the expression level of synapsin I S9 was decreased with no change in synaptic protein SNAP25 and PSD95 expression. **(B)** Statistical bar chart to show that CHIP, synapsin I, SNAP25, and PSD95 expression were present (*n* = 6, **p < 0.01, ***p < 0.001). **(C)** The dose of 5 μl Stub1-siRNA showed a decrease in the expression levels of ubiquitin and synapsin I and the phosphorylation levels of synapsin I S9, S427, and S605. **(D)** Statistical bar chart to show that ubiquitin, synapsin I, synapsin I S9, S427, and S605 expression were present (*n* = 6, *p < 0.05).

N2a cells are neuroblastoma from mouse tissues which contain endogenous CHIP expression. To further elucidate the correlation between decreased CHIP expression and synaptic protein expression, knock-down CHIP protein level was designed by siRNA in N2a cells. Three CHIP siRNA sequences named Stub1-siRNA were transfected. According to the manufacturer protocols, 5 μl Stub1-siRNA was able to achieve effective knock-down of CHIP expression (Figures 6A,C), and consequently the expression level of synapsin I S9 was decreased with no change in the expression levels of synaptic proteins SNAP25 and PSD95 (Figures 6A,C). In the dose of 5 μl Stub1-siRNA, we found the same protein expression changes as those in the hippocampus of ISO-15 group mice, which were the decreased expression levels of ubiquitin and synapsin I and the phosphorylation level of synapsin I S9, S427, and S605 (Figures 6B,C).

## Discussion

A substantial number of surgeries are performed annually in older adults, leading to their susceptibility to post-operative neuropsychiatric complications (Rengel et al., 2019). Epidemiologic studies showed that 11% of the world’s population was over 60 years of age; this was projected to increase, by 2050, to 22% of the population (Kanasi et al., 2016). To recapitulate the effect of inhalation anesthetic on aging, we used isoflurane-induced PND in 15-month-old mice in this study. Mice at this age might be considered to be in their early elderly stage (Lin et al., 2012). One minimum alveolar concentration (MAC, the concentration at which 50% of animals did not have a motor response to painful stimuli) of isoflurane in rodents was about 1.5% (Lin et al., 2012). Concentrations between 0.5 and 1.3 MACs were commonly used in clinical practice. The concentration of isoflurane was chosen to be 2% (1.3 MAC) to ensure the efficiency of anesthesia, with the heated pad for temperature control during the recovery.

The inhalation anesthetic isoflurane had been shown to increase Aβ accumulation and phospho-tau in rodent brains (Xie et al., 2008; Planel et al., 2009). In these findings, it had been proposed that brain neurodegeneration after anesthetic exposure contributed to the brain functional changes. Methods to probe behavioral responsiveness in animals were required to understand the mechanisms. Our study showed that old mice exposed to isoflurane displayed special learning impairment in five consecutive training days of MWM (the classic test for examining spatial learning and memory). However, after a 1-day interval, on the seventh day, no difference was illustrated; the long-term memory function was not affected after the 1-day interval. As for motor function, the performances were worse in gait function. As for motor function and anxiety-like behavior assessment, locomotor activity and willingness to explore in those aged mice received isoflurane exposure. However, we did not observe similar results in younger mice at the age of 12.5 months. Aging was one focus worthy of attention. Isoflurane induced cognitive, motor, and anxiety disturbances in an age-dependent manner. A reduced number of intersections and spine density was found by classical Golgi silver staining in the cortex neuron of old mice that received isoflurane exposure.

The UPS was highly specific in choosing the substrate proteins, which was possible by the sequential actions of three enzymes: E1, E2, and E3s (Collins and Goldberg, 2017). Ubiquitin activation was the first step mediated by E1, then ubiquitin was transferred from the E1 to the E2 enzyme and successively to the target protein *via* E3s (Passmore and Barford, 2004). Finally, misfolded, damaged, or unneeded cellular proteins were degraded (Thibaudeau and Smith, 2019). Active caspase-3, -7, and -9 were inhibited by the ubiquitin E3 ligase (Scott et al., 2005). The level of Cdh1 (E3 ubiquitin ligases) in the hippocampus was downregulated during isoflurane-induced neuroapoptosis (Li et al., 2017). The molecular chaperone and ubiquitin E3 ligase CHIP was a ubiquitously expressed cytosolic protein having dual function as both, which polyubiquitinate phosphorylated 4-repeat-Tau, alleviating tau aggregation and reversing neuronal toxicity (Sahara et al., 2005). In CHIP^–^/^–^ mice, neuronal caspase-3 levels and activity, as well as caspase cleaved tau immunoreactivity, were increased (Dickey et al., 2006). In our study, it was also found that the expression of CHIP was decreased in the cortex and hippocampus, with a reduced level of ubiquitin and apoptosis activation in isoflurane-exposed aged mice.

In the nervous system, to achieve synapse-specific effects, proteolysis needed to be spatially restricted. The UPS-associated proteins were abundant and essential for many aspects of neuronal function. Abundant evidences indicated that ubiquitin–proteasome-mediated degradation had a role in the molecular mechanisms underlying synaptic plasticity that operated in the nucleus as well as at the synapse (Hegde, 2004). Synaptic ubiquitin pools were particularly vulnerable to fluctuations in ubiquitin stability due to their remote location away from the site of ubiquitin synthesis (Chen and Sun, 2009; Chen et al., 2011). The UPS was closely associated with synaptic function indicating that ubiquitin conjugation was critical for efficient protein turnover at synaptic terminals. E3 ligases had been implicated in the turnover of presynaptic proteins such as RIM1 and synaptophysin (Wheeler et al., 2002; Yao et al., 2007). Neurotransmitter receptors had been shown to be internalized through a ubiquitin-dependent process (Buttner et al., 2001).

Inhalation anesthetics such as isoflurane modulated synaptic and extra-synaptic neurotransmission through multiple post-synaptic targets (Rudolph and Antkowiak, 2004). There was growing evidence that inhalation anesthetics target presynaptic mechanisms in addition to post-synaptic receptors (Baumgart et al., 2015; Zalucki et al., 2015; Bademosi et al., 2018). After adult male rats were exposed to 1.8% isoflurane for 2 h, isoflurane impaired hippocampal learning and modulated synaptic plasticity in the post-anesthetic period (Uchimoto et al., 2014). Isoflurane acted through multiple distinct pathways to inhibit neurotransmission, such as inhibiting synaptic vesicle (SV) exocytosis at nerve terminals *via* voltage-gated Ca^2+^ channels (Torturo et al., 2019), disrupting excitatory neurotransmitter dynamics *via* inhibition of the mitochondrial complex (Zimin et al., 2018), enhancing phasic inhibitory transmission *via* post-synaptic gamma-aminobutyric acid type A receptors while suppressing excitatory transmission through presynaptic mechanisms (Peters et al., 2008), and so on. Identifying presynaptic mechanisms of general anesthetics was critical to understanding their effects on synaptic transmission. Such alterations in the balance of excitatory to inhibitory transmission could result in reduced neuronal interactions and network-selective effects observed in the anesthetized central nervous system.

In this study, we found that declined CHIP expression in isoflurane-exposed aged mice could lead to decrease in synapse function-relevant protein synapsin I and phosphorylation, which was consistent with the changes in protein expression in CHIP knock-down cellular model. Synapsin I was the most abundant brain phosphoprotein present in conventional synapses of the central nervous system. It had been proposed that synapsin I in its dephosphorylated state may tether SVs to actin filaments within the cluster from where SVs were released in response to activity-induced synapsin phosphorylation (Shupliakov et al., 2011). Synapsin I mediated coupling release events to action potentials at the latest stages of exocytosis (Bykhovskaia, 2011). Decreased CHIP expression altered the function of the UPS and apoptosis, which might disturb many important functions of synaptic protein expression and phosphorylation. Hence, decreased expression level of synapsin I and phosphorylation level of synapsin I S9, S427, and S605 were found. However, there were no changes in the expression levels of synaptic proteins SNAP25 and PSD95. Typically, SNAP25 was a pre-synaptic protein, and PSD95 was a post-synaptic protein. It implied that alteration by decreased CHIP was specifically for some synaptic proteins, not all synaptic proteins. The dynamic synapsin phosphorylation/dephosphorylation level change could decipher the synaptic degeneration mechanism in the underlying pathogenesis of PND.

One limitation of our study was that we did not include mice at the same age of 15 months receiving lower concentration of isoflurane. One study found significant cell death, but the rats’ learning and memory functions were not shown in the brains of 16-month-old rats after isoflurane exposure (Stratmann et al., 2010). However, more findings seem to be different from that conclusion (Culley et al., 2004; Xie et al., 2008; Lin et al., 2012). We speculated that it may be due to the assessment of cell injury and cognitive functions at different time points or different isoflurane concentrations (Lin et al., 2012).

In this model, it had been shown that the ubiquitin E3 ligase CHIP was a promising target associated with the neurodegeneration in PND induced by isoflurane. And our finding strongly suggested that aging was the big important factor for isoflurane-induced alterations. The decline in CHIP protein expression altered synapsin expression and phosphorylation and might reveal the reason for synaptic degeneration in the underlying pathogenesis of PND. More studies on the upregulation of ubiquitin E3 ligase CHIP to rescue the UPS and synaptic function would need to be done in subsequent work.

## Data Availability Statement

The original contributions presented in the study are included in the article/supplementary material, further inquiries can be directed to the corresponding authors.

## Ethics Statement

The animal study was reviewed and approved by the Experimental Animal Committee at Tongji Hospital, Tongji Medical College, Huazhong University of Science and Technology.

## Author Contributions

QX and JX designed the research. QX, LX, and YW conducted the research. ML, QL, and TJ analyzed the data. QX, AL, and YZ drafted and revised the manuscript. All authors approved the final version to be submitted.

## Conflict of Interest

The authors declare that the research was conducted in the absence of any commercial or financial relationships that could be construed as a potential conflict of interest.

## Publisher’s Note

All claims expressed in this article are solely those of the authors and do not necessarily represent those of their affiliated organizations, or those of the publisher, the editors and the reviewers. Any product that may be evaluated in this article, or claim that may be made by its manufacturer, is not guaranteed or endorsed by the publisher.
